# Oncoplastic breast surgery with latissimus dorsi myocutaneous flap for large defect in patients with ptotic breasts: is it feasible when combined with local flaps?

**DOI:** 10.1186/1477-7819-12-65

**Published:** 2014-03-27

**Authors:** Seungju Lee, Jeeyeon Lee, Seokwon Lee, Youngtae Bae

**Affiliations:** 1 Department of Surgery, Busan Medical Center, 359 Worldcup-daero, Yeonje-gu, Busan 611-706, Republic of Korea; 2 Department of Surgery, Medical Research Institute, Pusan National University, Ami-dong 1-ga, Seo-gu, Busan 602-739, Republic of Korea

**Keywords:** Breast neoplasm, Ptotic breast, Latissimus dorsi myocutaneous flap, Combination of flap

## Abstract

**Background:**

The latissimus dorsi myocutaneous flap (LDMCF) is frequently applied to breast cancer patients for breast reconstruction. However, the LDMCF is considered inappropriate for patients with ptotic breast. The authors investigated combining LDMCF and two local flaps for large defects of the breast after partial mastectomy in patients with ptosis.

**Methods:**

Nineteen patients with breast cancer underwent a partial mastectomy with immediate reconstruction. Reconstruction methods consisted of LDMCF, thoraco-epigastric flap, and inferior pedicled rotational local flap, referred to as a combined pedicle flap. The cosmetic results were self-assessed after chemotherapy and radiotherapy by a four-point scoring system.

**Results:**

Ptosis was graded as follows: two patients with grade 1, 10 patients with grade 2, and seven patients with grade 3. The mean tumor size was 2.7 cm and multifocality was identified in 11 patients (57.9%). The mean excised volume was 468.5 cm^3^ and the percentage of excised volume was 46.2%. The cosmetic results were excellent in five patients, good in seven patients, fair in six patients, and poor in one patient.

**Conclusion:**

The combined pedicle flap, consisting of LDMCF, thoraco-epigastric flap, and inferior pedicled rotational local flap, allows good cosmesis in breast cancer patients with large breasts or ptosis despite a wide excision.

## Background

The latissimus dorsi myocutaneous flap (LDMCF) is a useful method as oncoplastic breast surgery. LDMCF can supply adequate volume and be easily acquired. Besides, it has an advantage of low complication rates. LDMCF is, however, considered inappropriate for patients with ptotic breast. Though the saline-filled prosthesis is combined with LDMCF, the natural shape of drooping breast cannot be achieved. Therefore, bilateral reduction mammoplasty is widely used for breast cancer patients with large or ptotic breast [1-5]. The location of the tumor, however, is the limiting factor of reduction mammoplasty. In addition, reduction mammoplasty alone cannot satisfy the cosmetic result for large defects. Is it, then, impossible to apply LDMCF to the breast cancer patients with ptotic breasts? We combined LDMCF, thoraco-epigastric flap (TEF), and inferior pedicled rotational local flap (IPRLF), as a ‘combined pedicle flap,’ for large defects in women with macromastia or ptotic breast.

## Methods

From July 2010 to November 2011, we performed partial mastectomy with a combination of LDMCF, TEF, and rotational local flap on 19 patients with ptotic breast. Ptosis was graded according to the Regnault classification (Table 1) [6]. Magnetic resonance imaging (MRI) of breast was performed in all patients, and the preoperative breast volume was obtained by three-dimensional reconstitution of MRI. The excised breast volume was calculated using the records of pathologic reports.

**Table 1:** Regnault’s classification of ptosis

Minor ptosis (1st degree)	Nipple at inframammary fold
Moderate ptosis (2nd degree)	Nipple below inframammary fold, but above lower breast contour
Severe ptosis (3rd degree)	Nipple below inframammary fold, and at lower breast contour
Glandular ptosis	Nipple above inframammary fold, but breast hangs below fold
Pseudoptosis	Nipple above inframammary fold, but breast is hypoplastic and hangs below fold

Indications for this combined pedicle flap were patients with ptotic breasts who refused bilateral reduction mammoplasty because of the scar on the contralateral breast. A combined pedicle flap was also planned when patients had more than 2 cm of breast cancer or less than 2 cm with multifocal breast cancer. In these cases, an immediate additional combination of flap was performed when the surgical margin was positive and further excision was required or the tumor size was larger than the preoperative evaluation or the defect could not be filled with LDMCF only.

Patients who had scars on the back, or previous surgery in the axilla region were excluded in this study. Patients were asked to rate their cosmetic results using a scale of 1 to 4 after chemotherapy and radiotherapy.

This study was retrospectively reviewed and approved by the Institutional Review Board, and all patients provided written informed consent.

### Surgical technique

First, we must obtain LDMCF to replace the defect of the breast. In a lateral supine position, the donor site skin incision was elliptical, ranged from 5 to 7 cm wide and from 15 to 18 cm long. The obtained LDMCF was put in a subcutaneous pocket in the posterior axillary fold. The skin flap was fixed using monosyn 3-0 sutures 4 cm apart started at the base of the upper flap and at the inferior limit of the LD resection in relation to the iliac crest.

**Figure 1:**
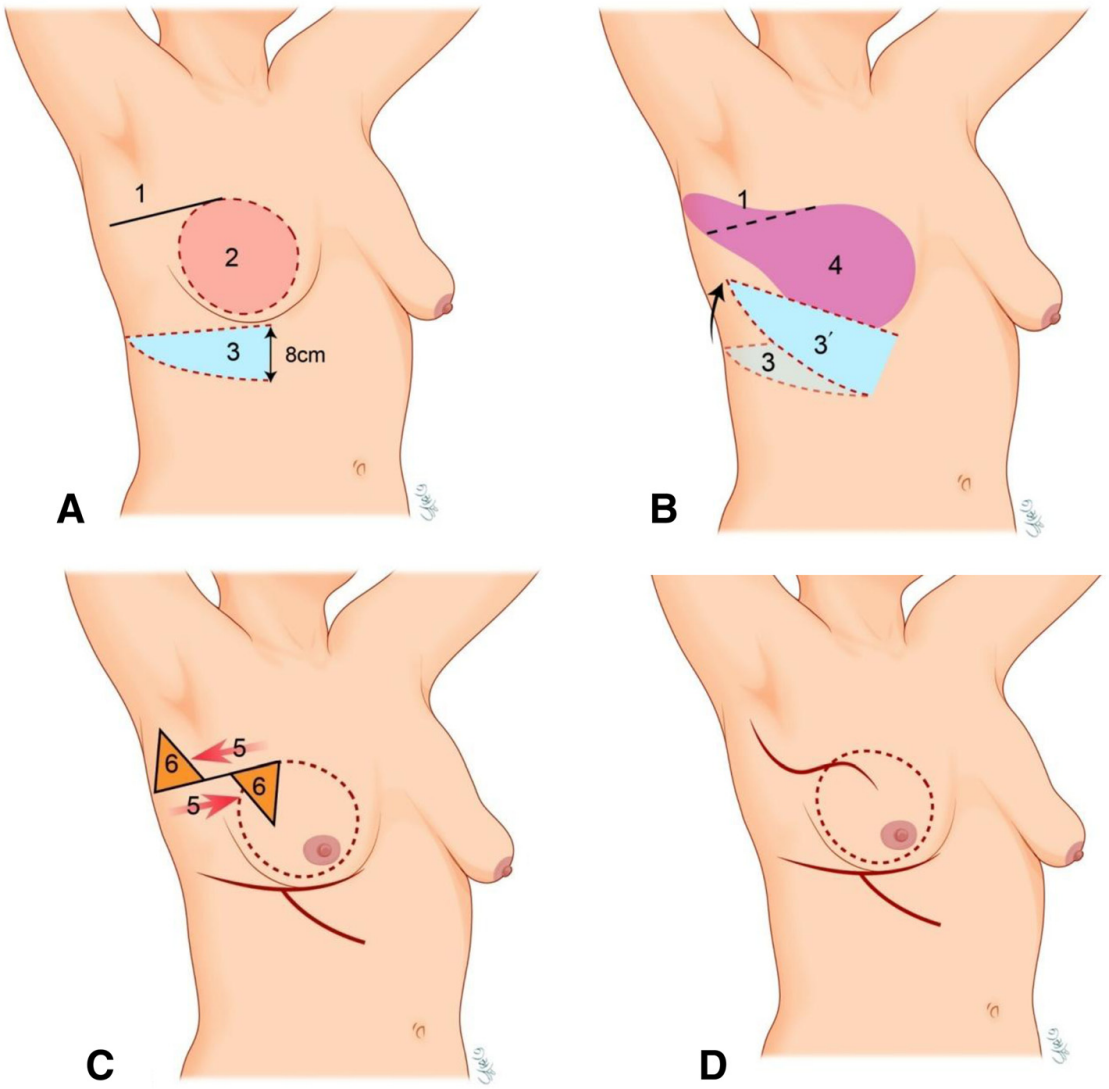
**Combined pedicle flap surgical technique. (A)** Through an incision (1), the partial mastectomy was done (2). The width of TEF (3) should be more than 8 cm. **(B)** The obtained TEF placed at the lower outer border of LDMCF (3’). Defect from partial mastectomies would be filled with LD flap (4) and TEF. **(C)** The counter traction (5) of the inferior pedicled rotational local flap was done and the ‘dog ear’ (6) was trimmed off both sides of the incision. **(D)** Completion of the combined pedicle flap.

The patient was then placed in a supine position with both arms abducted. The line of the IPRLF was incised from the mid-axillary line to the tumor location, following the skin crease (Figure 1A). Through this incision, a sentinel lymph node biopsy was done if needed and the tumor was resected with 1-2 cm of the margin to ensure oncologic safety (Figure 1A). A circumferential intraoperative frozen biopsy was done on the remnant breast tissue, and additional breast tissue was resected when the frozen biopsy results were positive. The posterior axillary line was then dissected and the LDMCF was passed under the tunnel. The island skin of LDMCF was de-epithelialized thinly, and the LDMCF was inserted into the place where the breast tissue was removed. The TEF was designed with a width greater than 8 cm (Figure 1A). The TEF should be obtained with skin, subcutaneous fat, and the anterior serratus muscle fascia. The superior epigastric vessels and perforators must be preserved because these are the vascular supply of this flap. The skin of TEF was de-epithelialized, and the prepared TEF was transposed under a tunnel of breast parenchyma from the inframammary fold to the lower outer region of breast defect (Figure 1B). Large defects from partial mastectomies would be completely filled with LDMCF and TEFs. The counter traction of IPRLF was done and the ‘dog ear’ was removed on the both sides of the incision (Figure 1C). After placing a drain in the defect, the wounds were closed, using 3-0 and 4-0 absorbable monofilament sutures. The drain was removed when the volume drained was less than 50 cc.

## Results

The mean age of the 19 patients was 49.6 years (range, 32 to 69 years). Two patients had grade 1 ptosis, 10 patients had grade 2 ptosis, and seven patients had grade 3 ptosis. The mean initial tumor size on preoperative evaluation was 2.4 cm (range, 1.1 to 5.0 cm), and the mean pathologic tumor size was 2.7 cm (range, 0.8 to 8.0 cm). The location of the tumor was as follows: upper outer quadrant (n = 7); upper central area (n = 3); lower outer quadrant (n = 4); lateral area (n = 2); medial area (n = 1); central area (n = 1); and upper inner quadrant (n = 1). Eleven cases had multifocal breast cancer. The mean excised breast volume was 468.5 cm^3^ (range, 105.0 to 1,734.0 cm^3^) and the mean percentage of excised breast volume was 46.2% (range, 19.6 to 97.3%). The mean duration of surgery was 269.3 min (range, 135.0 to 345.0), and the mean hospital stay was 12.4 days (range, 7 to 27 days). Patients’ pathologic stages were as follows: ductal carcinoma *in situ* (n = 1); I (n = 1); IIA (n = 7); IIB (n = 7); IIIA (n = 1); and IIIC (n = 2). Wound dehiscence occurred in two cases. These complications resulted from poor vascular supply at the TEF donor site (Table 2). These were resolved with conservative management and wound revision.

**Table 2:** Characteristics of the 19 patients who underwent combined pedicle flap procedure

**Patient no.**	**Ptosis grade**	**Age (years)**	**Pathologic tumor size (cm)**	**Location of tumor**	**Excised breast volume (cm**^**3**^**)**	**Excised breast percentage (%)**	**Tumor stage**	**Cosmetic result**
1	3	60	2.5	Upper central	381.9	42.7	IIA	Fair
2	3	46	3.2	Medial	378.6	27.6	IIB	Fair
3	3	69	1.3	Upper outer	261.9	22.4	IIA	Excellent
4	2	53	8.0	Upper outer	972.0	61.1	DCIS	Excellent
5	2	38	2.0	Upper outer	160.7	21.6	IIA	Good
6	1	43	2.5	Lower outer	345.0	53.9	IIB	Excellent
7	2	51	1.6	Upper outer	465.0	36.0	IIA	Good
8	3	37	4.0	Upper inner	1,734.0	97.3	IIB	Poor
9	2	50	2.1	Upper central	1,080.3	67.5	IIB	Fair
10	2	59	3.1	Lower outer	324.0	32.0	IIIA	Good
11	2	58	2.4	Lateral	341.0	53.0	IIB	Good
12	1	32	2.2	Upper outer	180.0	54.8	IIB	Excellent
13	3	38	2.5	Lower outer	330.0	46.3	IIB	Fair
14	2	48	0.8	Upper central	198.0	73.3	I	Fair
15	3	53	3.2	Lateral	815.2	87.2	IIIC	Good
16	2	45	3.7	Lower outer	105.0	21.4	IIA	Fair
17	2	52	2.5	Central	450.0	36.7	IIA	Good
18	3	52	2.2	Upper outer	178.0	22.5	IIIC	Good
19	2	58	2.2	Upper outer	201.3	19.6	IIA	Excellent
Mean		49.6	2.7		468.5	46.2		

Cosmetic outcomes were self-reported to be excellent in five cases (26.3%), good in seven cases (36.8%), fair in six cases (31.6%), and poor in one case (5.3%; Table 3).

**Table 3:** Cosmetic outcomes of the 19 patients who underwent combined pedicle flap surgery

**Cosmetic outcome**	**Patients (n,%)**
Excellent	5 (26.3)
Good	7 (36.8)
Fair	6 (31.6)
Poor	1 (5.3)

## Discussion

The application of oncoplastic surgery depends on the tumor size, tumor location, ratio of tumor to whole breast volume, and range of excision volume [7-12]. There are, therefore, a number of possible oncoplastic techniques for each case. LDMCF is a useful method of replacing defects caused by resection of breast tissue. Generally, the LDMCF is best suited for small or moderate sized breasts [13-17]. However, LDMCF will require additional technique, such as mastopexy or reduction mammoplasty, in the contralateral breast to obtain symmetry, when it is used for patients with large or ptotic breasts. Although the LDMCF is best suited for cases where tumors are situated in the upper, central, or lateral quadrants of the breast, in our study of nine patients, the tumor was located in the outer quadrant area and another six were in the upper inner quadrant area. For evaluation of possibility of immediate postoperative asymmetry and volume difference between both breasts during surgery, we have performed temporary skin closure after partial mastectomy defect replaced with LDMCF. The possibility will be increased in lean patients with thin LDMCF. In case there is a possibility of this and further deterioration in long-term follow-up after radiotherapy including fibrosis and becoming smaller than contralateral breast, we have planned combined flaps in these cases, instead of only LDMCF.

In addition, it is difficult to predict the volume of LDMCF; we occasionally encounter some cases in which LDMCF is insufficient for large defects. The combination of LDMCF and implants could fill a large defect. There is, however, a high risk of complications, such as capsular fibrosis and contracture of the adjacent tissue, in the late course after reconstruction with the LDMCF in combination with a prosthesis [18-20]. When the implant cannot be fixed in the appropriate place, the cosmetic result will be poor. Patients would feel a foreign body sensation if a prosthesis implant is used instead of the autologous tissue.

Reduction mammoplasty can be useful in patients with macromastia or ptosis. But the location of the tumor is the limiting factor of reduction mammoplasty. When the tumor is located in the upper inner quadrant, reduction mammoplasty cannot be an appropriate method [2,3]. Reduction mammoplasty is also limited to tumor location in the lower outer quadrant. The superior pedicle, which could lead to a poor cosmetic result, is applied to the tumor in that area [1,4,5]. Besides, the scar of the other breast might be annoying to the patients.

**Figure 2:**
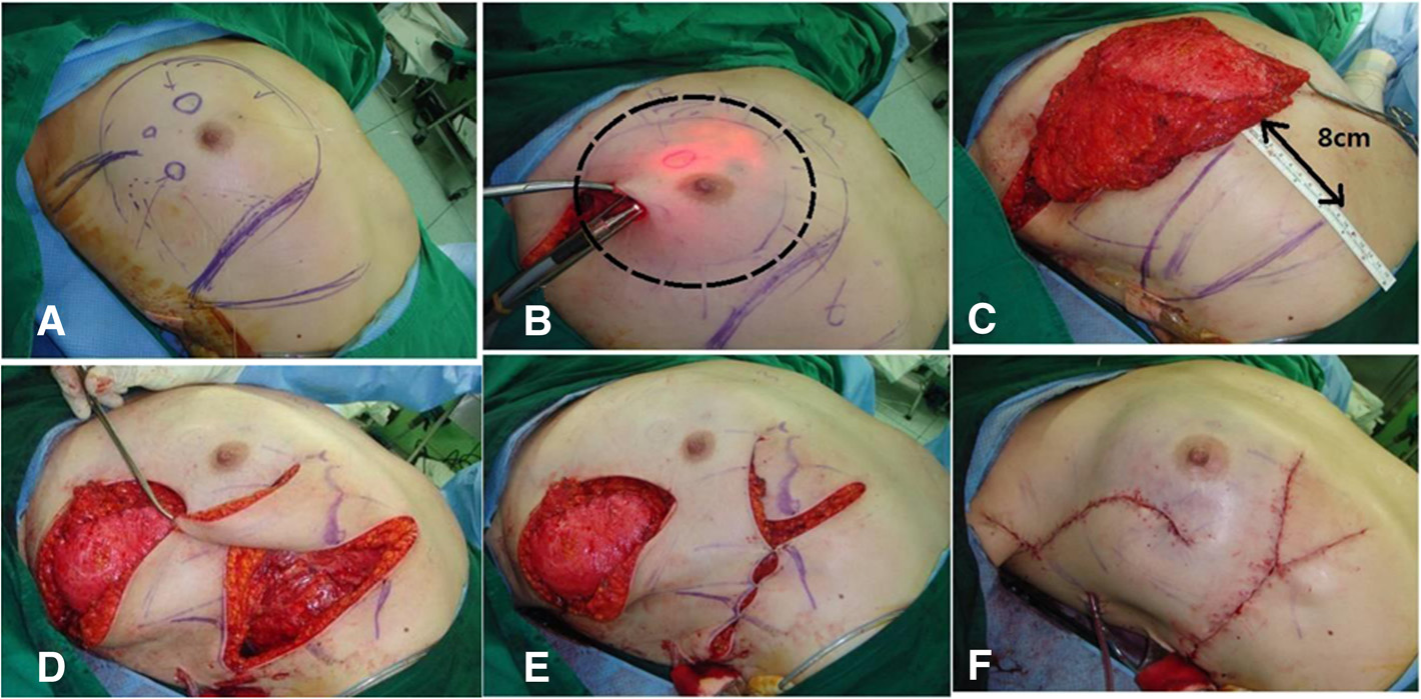
**Patient with multifocality. (A)** Preoperative view with marking of the skin incision. **(B)** Defect (dot circle) by transillumination after partial mastectomy. **(C)** Skin incision marking for TEF. The width of TEF should be more than 8 cm. **(D)** TEF would be rotated to lower outer margin of LDMCF. **(E)** Combination of LDMCF and TEF is filling the defect. **(F)** Skin closure after combined pedicle flap.

Our technique, which consists of LDMCF, TEF, and IPRLF, had no limitation of tumor location and could obtain good cosmetic results despite of wide excision. As shown in Figure 2, patient 17 had been diagnosed with breast cancer in the central region. She had multiple daughter cells in the upper inner site around the main tumor mass. We had to resect more breast tissue to obtain oncologic safety; therefore, we tried combined pedicle flaps in this patient. Her tumor stage was IIA and the percentage of excised breast volume was identified as 36.7%. As shown in Table 2, the cosmetic result of this case was good. And we had six more patients with positive margin during the operation, and their cosmetic results were also good.

Generally, excision of more than 20% of breast volume predicts poor cosmetic result [21,22]. In our study, the mean percentage of excised volume was 46.2%; therefore, this is a remarkable result with which to obtain oncologic safety. When the cancer or atypical ductal hyperplasia was identified in the intraoperative frozen section, the breast tissue excision was larger than the presumed volume. The combination of LDMCF, TEF, and rotational local flap would resolve this problem.

**Figure 3:**
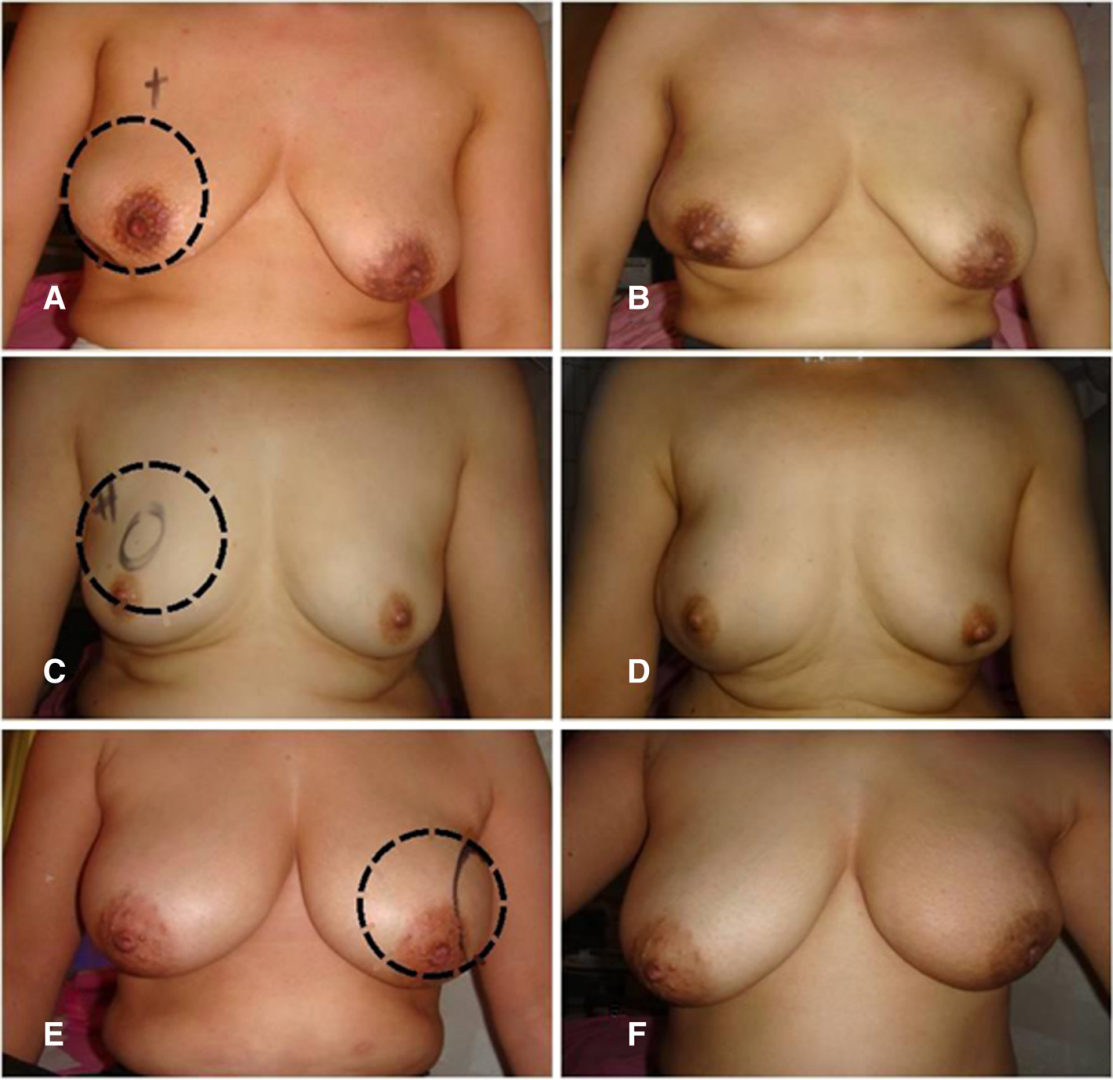
**Outcomes of combined pedicle flap. (A, C, E)** Preoperative views marked with breast cancer locations (dot circle). **(B, D, F)** Postoperative views after radiotherapy.

In ptotic breast patients, the upper chest region is flat and the glandular portion is drooping around the inframammary line. IPRLF could reduce the requirement of volume in the upper chest region, and fix the LDMCF from medial to lower region of the breast defect. When the LDMCF is passed through the tunnel, beneath the skin bridge separating the mastectomy and donor site, the mid axillary region may be bulging with this LD flap. This bulging of LDMCF could cause discomfort to patients after surgery. The IPRLF could help LDMCF transpose to the appropriate position without bulging because the counter traction of IPRLF can remove the tunnel space (Figure 1C). The Nipple-areolar complex (NAC) is often deviated upward when the LDMCF is only used in the volume replacement. To prevent this, we modified the combination of local flaps to displace the NAC downward [23]. The angle of rotation of TEF was obtuse to place the TEF at the lower outer border of LDMCF (Figure 1B). This combined pedicle flap, therefore, could achieve natural shape of the breast in ptotic breast patients. In our study, cosmetic outcomes were evaluated at least 6 months postoperatively. Although the number of this study was small, cosmetic results were generally acceptable, as shown in Figure 3.

As we mentioned in our previous study [23], the inferior scar of TEF is not visible on the front view and it creates a neo-inframammary line (Figure 1D). This is an advantage of this procedure compared to the scar of the contralateral breast in the reduction mammoplasty.

Although the mean operative time was 269.3 minutes, it can be shortened with surgical experience.

In our experience, some complications occurred, such as wound dehiscence caused by fat necrosis. In the case of wound dehiscence, it resulted in a relatively long hospital stay and poor cosmetic result. To prevent this complication, we designed the width of TEF as over than 8 cm and obtained TEF meticulously, using a Mayo scissor (Figure 1A). The use of Mayo scissor could avoid the thermal injury of electrocautery on the vascular supply of this flap.

## Conclusion

Combination of TEF and IPRLF could reduce the requirement of volume when LDMCF is insufficient for the large defects in patients, especially reluctant to the scar of the contralateral breast. The combined pedicle flap allows oncologic safety and good cosmetic results in breast cancer patients with large breasts or ptosis, despite a wide excision.

## Abbreviations

IPRLF: inferior pedicled rotational local flap; LDMCF: latissimus dorsi myocutaneous flap; MRI: Magnetic resonance imaging; NAC: Nipple-areolar complex; TEF: thoraco-epigastric flap.

## Competing interests

The authors declare that they have no competing interests.

## Authors’ contributions

SL, general surgeon who participated in data collection, design of the study and drafted the manuscript. JL, general surgeon who participated in surgical procedures. SL, general surgeon who participated in surgical procedures. YB, general surgeon who carried out the surgical procedures and contributed to study conception and critical revision. All authors read and approved the final manuscript.
